# Pancreatic Tuberculosis or Autoimmune Pancreatitis

**DOI:** 10.1155/2014/410142

**Published:** 2014-04-15

**Authors:** Ayesha Salahuddin, Muhammad Wasif Saif

**Affiliations:** ^1^Miller School of Medicine, University of Miami, 1120 NW 14th Street, Suite 1185, Miami, FL 33136, USA; ^2^Exp. Therapeutics, Tufts University School of Medicine, 800 Washington Street, Boston, MA 02111, USA

## Abstract

*Introduction*. Isolated pancreatic and peripancreatic tuberculosis is a challenging diagnosis due to its rarity and variable presentation. Pancreatic tuberculosis can mimic pancreatic carcinoma. Similarly, autoimmune pancreatitis can appear as a focal lesion resembling pancreatic malignancy. Endoscopic ultrasound-guided fine needle aspiration provides an effective tool for differentiating between benign and malignant pancreatic lesions. The immune processes involved in immunoglobulin G4 related systemic diseases and tuberculosis appear to have some similarities. *Case Report*. We report a case of a 59-year-old Southeast Asian male who presented with fever, weight loss, and obstructive jaundice. CT scan revealed pancreatic mass and enlarged peripancreatic lymph nodes. Endoscopic ultrasound-guided fine needle aspiration confirmed the presence of *mycobacterium tuberculosis*. Patient also had high immunoglobulin G4 levels suggestive of autoimmune pancreatitis. He was started on antituberculosis medications and steroids. Clinically, he responded to treatment. Follow-up imaging showed findings suggestive of chronic pancreatitis. *Discussion*. Pancreatic tuberculosis and autoimmune pancreatitis can mimic pancreatic malignancy. Accurate diagnosis is imperative as unnecessary surgical intervention can be avoided. Endoscopic ultrasound-guided fine needle aspiration seems to be the diagnostic test of choice for pancreatic masses. Long-term follow-up is warranted in cases of chronic pancreatitis.

## 1. Introduction 

Isolated primary pancreatic and peripancreatic tuberculosis (TB) is rare even in endemic areas. Pancreatic tuberculosis has diverse clinical presentation. Patients can present with acute pancreatitis, gastrointestinal bleeding, or obstructive jaundice. This entity can also present as an isolated pancreatic mass mimicking pancreatic carcinoma. Its rarity and variable clinical presentation makes pancreatic TB a very challenging diagnosis. A few cases of coexisting pancreatic carcinoma and tuberculosis have also been reported in the literature [1, 2]. Autoimmune pancreatitis and chronic pancreatitis can also present as a pancreatic mass. We present a case report of a patient with peripancreatic tuberculosis. This case has some features consistent with autoimmune pancreatitis based on the consensus diagnostic criteria for autoimmune pancreatitis published by International Association of Pancreatology in 2011 [3] and review of the relevant literature.

## 2. Case Presentation

**Figure 1:**
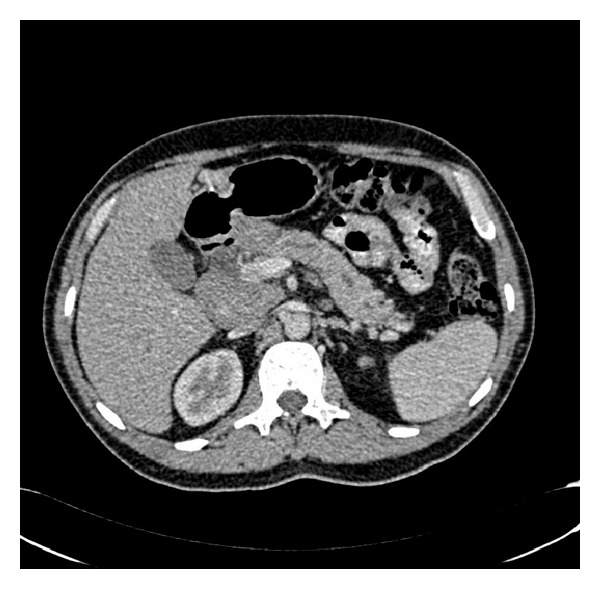
Initial presentation. Periampullary (pancreatic head) mass and dilated pancreatic duct.

Our patient is a 59-year-old Southeast Asian male with past medical history significant for gastroesophageal reflux disease, hiatal hernia, and hyperlipidemia. He initially presented with low-grade fevers, generalized weakness associated with decreased appetite and weight loss. Subsequently, he noticed dark discoloration of the urine. He did not take any medications. He was a lifetime nonalcoholic and used to smoke occasionally (1-2 cigarettes/week). Family history was significant for diabetes mellitus, hypertension, myocardial infarction, and stroke. Initial laboratory work showed elevated liver enzymes and raised amylase, lipase (Table 1). Ultrasound and computerized tomography (CT) of the abdomen showed dilated common bile duct, dilated pancreatic duct, and enlarged lymph nodes in the porta hepatis, peripancreatic and perigastric regions. A hypoechoic mass measuring 3 by 4 cm was seen in the pancreatic head. There was a strong suspicion of pancreatic carcinoma (Figure 1).

**Figure 2:**
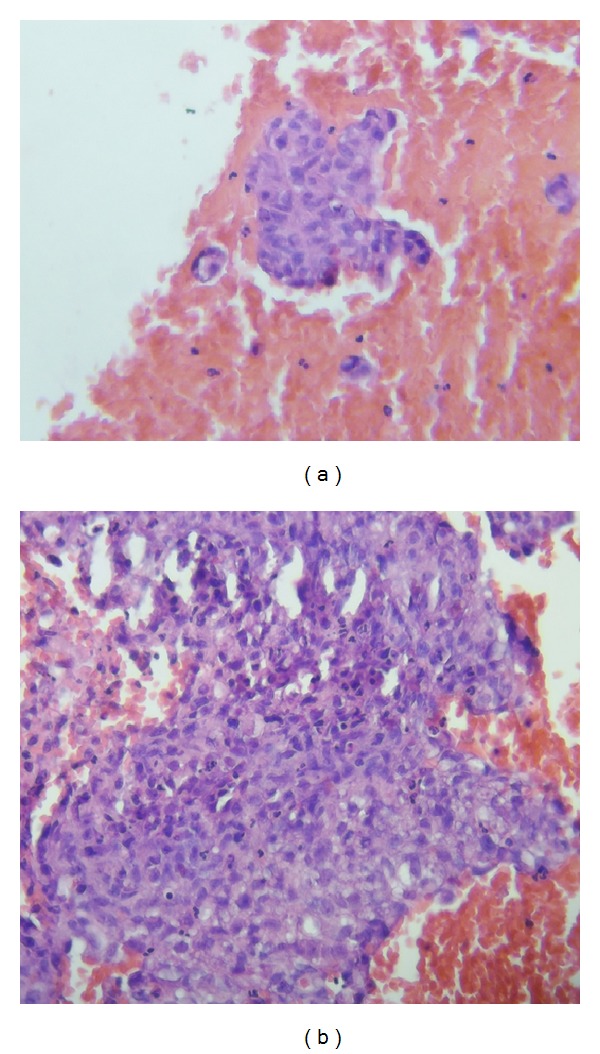
(a) and (b) Hematoxylin and eosin stain of the peripancreatic lymph node showing epithelioid granuloma.

Endoscopic ultrasound- (EUS-) guided fine needle aspiration (FNA) of the pancreatic mass showed reactive atypia with no evidence of malignancy and celiac lymph node FNA cytology showed atypical cells with granuloma (Figures 2(a) and 2(b)). Quantiferon gold came back positive and* mycobacterium tuberculosis* (MTB) DNA was detected via polymerase chain reaction (PCR) from the biopsy specimen. Tumor markers including CA19-9 (carbohydrate antigen 19-9), AFP (alpha-fetoprotein), CA 125 (cancer antigen 125), and CEA (carcinoembryonic antigen) were normal. Angiotensin converting enzyme level was also within normal limits. Subsequently, the patient developed jaundice associated with fever. Endoscopic retrograde cholangiopancreatography (ERCP) was done and a biliary stent was placed in the common bile duct. Biopsy from the distal common bile duct also showed granulomatous inflammation. Patient was started on antituberculosis treatment (ATT) with rifampin 600 mg, ethambutol 1200 mg, isoniazid 300 mg, and pyrazinamide 1500 mg, which he tolerated well. IgG4 came back positive at 0.5 g/L (0.25 g/L–0.318 g/L) leading to suspicion of autoimmune pancreatitis. He was also started on prednisolone 40 mg orally every day. One month after the initiation of treatment, a repeat CT scan of the abdomen did not show any changes in the size of the pancreatic head mass or lymph nodes.

**Figure 3:**
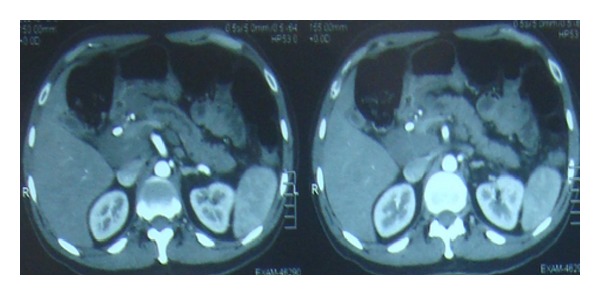
Six months after initial presentation. Persistent enlargement of peripancreatic lymph nodes and pancreatic duct dilatation.

Six months after initial presentation, he developed an episode of acute pancreatitis. By this time he was on ATT for five months and prednisolone for four months. CT scan showed increase in the size of lymph nodes and persistent common bile duct and pancreatic duct dilatation (Figure 3). Another ERCP was performed with stent exchange and retrieval of few gallstones. Pancreatic mass and the pancreatic duct could not be reached. EUS-guided biopsy of the lymph node showed reactive hyperplasia and was negative for malignancy or granuloma. MTB DNA was negative. Ampullary tissue biopsy also showed inflammatory changes. Magnetic resonance cholangiopancreatography (MRCP) was also performed which showed persistent enlarged lymph nodes and enlarged pancreatic duct but some regression of pancreatic mass.

**Figure 4:**
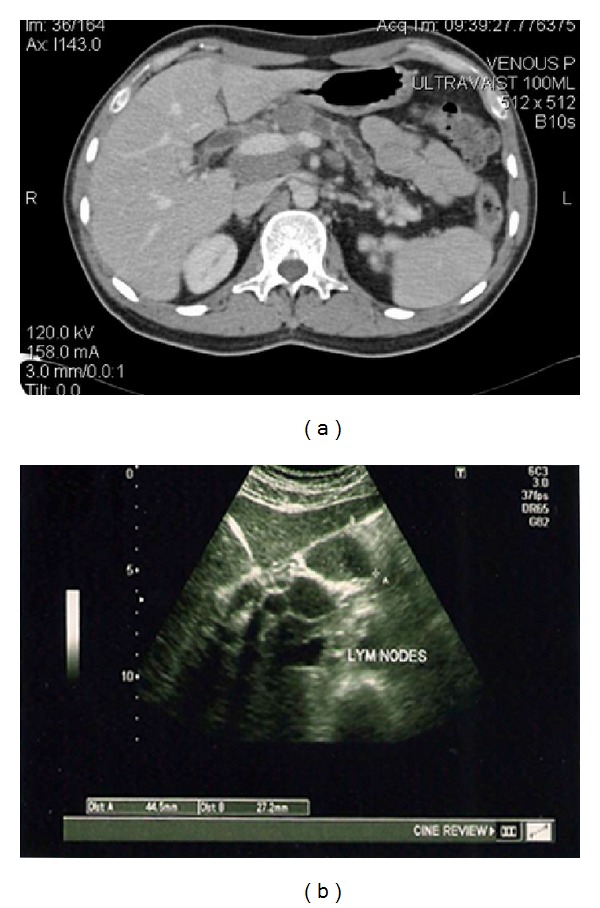
18 months after initial presentation. (a) CT showing persistent enlarged peripancreatic lymph nodes, pancreatic atrophy, and dilatation of pancreatic duct. (b) Ultrasound abdomen showing enlarged peripancreatic lymph nodes.

Patient completed a total of eighteen months of ATT and was tapered off steroids as well. Follow-up laboratory tests showed normal liver function test, amylase and lipase level (Table 1). IgG4 levels also decreased to a normal range (Table 1). He had a few episodes of self-resolving nausea and abdominal pain. Imaging studies including CT, ultrasound, and MRCP repeated after completing ATT and steroids continued to show enlarged lymph nodes in the porta hepatis, peripancreatic regions, dilated CBD, and pancreatic duct with atrophic looking pancreas (Figures 4(a) and 4(b)). At present, patient is doing well with minimal symptoms and is able to maintain weight.

## 3. Discussion

The gastrointestinal tract is the sixth most common site of extrapulmonary involvement for tuberculosis [4]. Incidence of abdominal tuberculosis is about 11–16% [5]. Isolated primary pancreatic TB is very rare even in endemic areas. The presence of pancreatic enzyme appears to confer resistance to invasion of* Mycobacterium tuberculosis *[6, 7]. Incidence of associated active pulmonary disease is quite variable and has been reported to be as high as 29% [8]. Men and women are affected equally [9]. In a MEDLINE search of English language articles from 1966 to 2004, 116 cases of pancreatic tuberculosis were identified [10]. From 2005 till now based on PUBMED search using the MeSH terms “Tuberculosis” and “Pancreas” including literature from English and other languages we have identified 49 case reports and 11 case series which include about 164 patients.

Postulated mechanisms by which* Mycobacterium tuberculosis* bacilli reaches the gastrointestinal tract include hematogenous spread from the pulmonary focus, ingestion of bacilli from the sputum in case of active pulmonary disease, and direct spread from an adjacent organ or lymphatic spread [4, 11]. Lymphohematogenous dissemination from an occult lung focus seems to be the most common cause [7, 12].

Abdominal pain is the most common symptom; other constitutional symptoms include anorexia, weight loss, fever, and night sweats. Cases of obstructive jaundice [13, 14], gastrointestinal bleeding [15], acute pancreatitis [16], portal hypertension [16, 17], and pancreaticobiliary fistula [18] with pancreatic TB have been reported. Most common location of mass in pancreatic TB is the head or the body of the pancreas [19], but isolated involvement of tail has also been reported [20].

Most cases have high erythrocyte sedimentation rate and skin test is positive in about 70% cases [10]. In our case the ESR value was 95 mm/hour and skin test was negative. Definitive diagnosis can only be achieved by histological confirmation. The success rate of identifying acid-fast bacilli from the biopsy specimen has been between 20 and 40% [7, 9]; cultures were found to be positive in about 77% cases [7]. PCR has been found to be significantly superior to smear and culture in detecting MTB and also provides rapid results [21]. Drug susceptibility cannot be assessed with PCR and another limitation is region specific variation in the genome of mycobacterium.

Tuberculous lymph nodes are enlarged and can be conglomerated. On ultrasound the enlarged lymph nodes contain a central hypoechoic area, whereas on enhanced CT the central liquefied substance has low attenuation and peripheral inflammatory lymphatic tissue has higher attenuation. Pancreatic TB can appear as well-defined hypoechoic lesion on ultrasound and as a hypodense lesion on CT scan [22]. On MRI, T1 weighted fat suppressed images, pancreatic tuberculosis lesion appears hypointense, whereas on T2 weighted images it shows heterogeneous signal intensities [23].

EUS-guided FNA is known to be quite effective in diagnosis and staging of pancreatic carcinoma [24]. Ultrasound/endoscopic guided FNA cytology has emerged as a reliable and cost effective way of diagnosing pancreatic or peripancreatic TB [19, 25, 26].

Based on international consensus diagnostic criteria for autoimmune pancreatitis [3], some features of this case are similar to autoimmune pancreatitis type 1. These include age of presentation, obstructive jaundice as the initial presentation, and elevated IgG4 levels. The infiltration of IgG4 producing plasma cells was not reported. Follow-up scans done about 18 months after initial presentation showed pancreatic atrophy with pancreatic duct dilatation which is consistent with chronic pancreatitis [27]. Progression to chronic pancreatitis (CP) has not been reported with TB, but AIP can progress to CP. A retrospective study done by Maruyama et al. showed that about 22% of AIP patients would progress to CP [28]. Pancreatic head swelling and main pancreatic duct nonnarrowing were found to be two independent risk factors for chronic pancreatitis [28].

A few cases of IgG4 related systemic disease and concurrent mycobacterium infection have been reported in the literature. Kawano et al. reported a case of IgG4 related chronic sialadenitis and dacryoadenitis in a patient who had been treated for cervical lymph node tuberculosis [29]. Imai et al. reported a case of IgG4 related tubulointerstitial nephritis after remission of urinary tract tuberculosis [30]. To the best of our knowledge, case of coexisting autoimmune pancreatitis and pancreatic TB has not been reported.

There are some similarities in the immune phenomenon of IgG4 related diseases and tuberculosis infection, but this needs further investigation. Naturally occurring CD4+CD25+ regulatory T cells (Tregs) were found to be increased in peripheral blood and disease site of the patients with active TB as evident by increased expression of cell surface CD25 and FoxP3mRNA [31, 32]. Tuberculosis has also been shown to promote T helper-2 (Th 2) cell differentiation via IL-1*β* induction in the dendritic cells [33]. A study by Zen et al. found high concentration of Th2 cytokines and Tregs in tissues affected by IgG4 related sclerosing pancreatitis and cholangitis [34].

Follow-up with radiological imaging was very important in this case. Most of the cases of pancreatic TB reported in the literature did not have any long-term sequel after successful treatment with antituberculosis treatment. Kim et al. conducted a retrospective analysis of 42 patients diagnosed with peripancreatic tuberculosis; in their analysis only one patient showed progressive disease based on imaging criteria [35]. In cases of AIP, increased risk of cancer [36–38] and pancreatic duct stone formation [36] has been reported.

Despite strong suspicion of pancreatic tuberculosis, this case maintains its obscurity. Incomplete resolution of radiological findings, slower than the expected response to ATT and progression to chronic pancreatitis, forces one to think of other possible pathological processes. Autoimmune pancreatitis remains as a differential diagnosis, but infiltration of IgG4 producing plasma cells was not checked. Early diagnosis is imperative in both cases as unnecessary surgical intervention can be avoided.

## Figures and Tables

**Table 1:** 

Blood tests (Normal ranges)	Initial presentation	Five months later	Seven months later	Twelve months later
ALT (9–40 U/L)	310 U/L	34 U/L	31 U/L	19 U/L
Total bilirubin (0.2–1.2 mg/dL)	3.9 mg/dL	0.8 mg/dL	1.0 mg/dL	1.0 mg/dL
AP (65–306 U/L)	2370 U/L	79 U/L	72 U/L	79 U/L
Amylase (25–125 U/L)	245 U/L	497 U/L	2045 U/L	89 U/L
Lipase (13–60 U/L)	352 U/L	805 U/L	2020 U/L	100 U/L
IgG 4 (0.25–0.3128 g/L)	0.5 g/L	0.3 g/L	0.262 g/L	Not checked

ALT: alanine aminotransferase, AP: alkaline phosphatase, IgG 4: immunoglobulin G4.
